# Supplementary material for: Structural and Functional Analysis of ASFV pI73R Reveals GNB1 Binding and Host Gene Modulation
Source: Int J Mol Sci. 2025 Dec 5;26(24):11768. doi: 10.3390/ijms262411768 (PMC12733259; doi:10.3390/ijms262411768)
Supplement: Supplementary file 1 [file ijms-26-11768-s001.zip › Suppl. Figures_I73R_v3-IJMS-submission-final-1.pdf]

Supplementary Figures

Structural and Functional Analysis of ASFV pI73R Reveals GNB1 Binding and Host Gene Modulation

Katarzyna Magdalena Dolata [1,*], Barbara Bettin [1], Richard Küchler [1], Katrin Pannhorst [1], Dmitry S. Ushakov [1], Walter Fuchs [1], Axel Karger [1,*]

[1] Institute of Molecular Virology and Cell Biology, Friedrich-Loeffler-Institut, Federal Research Institute for Animal Health, Südufer 10, 17493 Greifswald-Insel Riems, Germany

* Correspondence: katarzyna.dolata@fli.de (K.M.D.); axel.karger@fli.de (A.K.); Tel.: +49 38351 7 1535 (K.M.D.); +49 38351 7 1247 (A.K.)

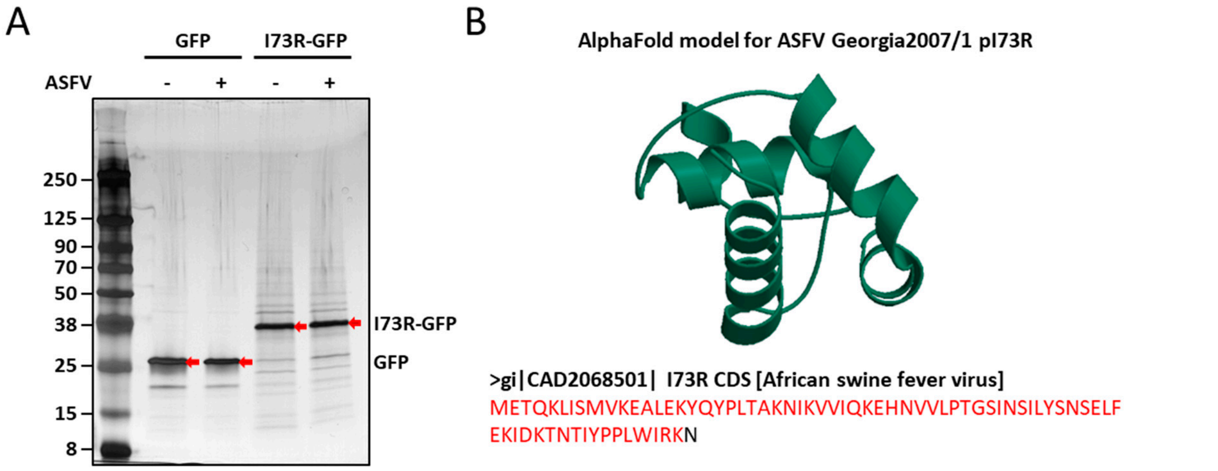

Gene	Protein name	Mass (kDa)	Protein score	Unique peptides	Seq. coverage
**I73R**	Uncharacterized protein I73R	8.52	323.31	14	98.6 %

Figure S1. Confirmation of ASFV I73R expression in WSL cells and mass spectrometric detection in immunopurified samples. (A) Immunoblot showing coimmunoprecipitates of GFP protein (control) and GFP-tagged viral protein I73R in WSL non- infected and ASFV-infected cells. GFP and GFP-I73R were detected by using anti-GFP antibody. Masses are shown in kilodaltons (kDa). (B) I73R protein sequence coverage (in red) and identification parameters using mass spectrometry.

WSL
non-infected

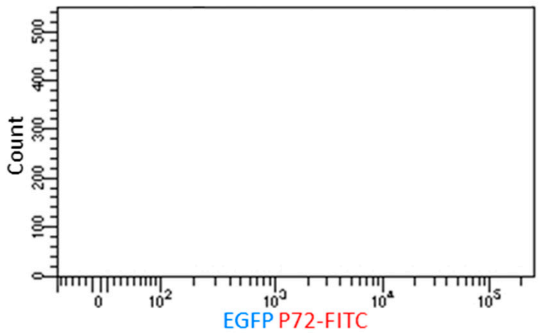

Population	#Events	%Parent	%Total
All Events	20,000	####	100.0
Cells	19,710	98.6	98.6
Singlets	16,849	85.5	84.2
single cells	16,465	97.7	82.3
ASFV+	0	0.0	0.0
EGFP+	0	0.0	0.0
P72+	0	####	0.0
ASFV+ EGFP+	0	0.0	0.0

WSL
ASFV-infected

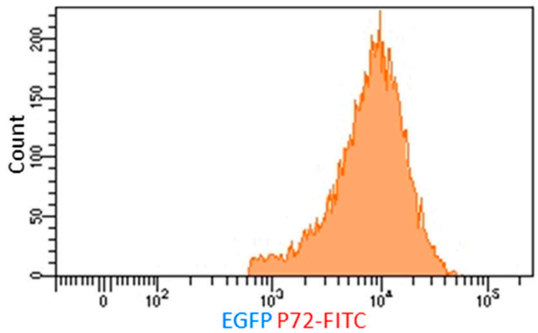

Population	#Events	%Parent	%Total
All Events	20,000	####	100.0
Cells	19,483	97.4	97.4
Singlets	15,895	81.6	79.5
single cells	15,465	97.3	77.3
ASFV+	8,079	52.2	40.4
EGFP+	0	0.0	0.0
P72+	0	####	0.0
ASFV+ EGFP+	0	0.0	0.0

WSL GFP
non-infected

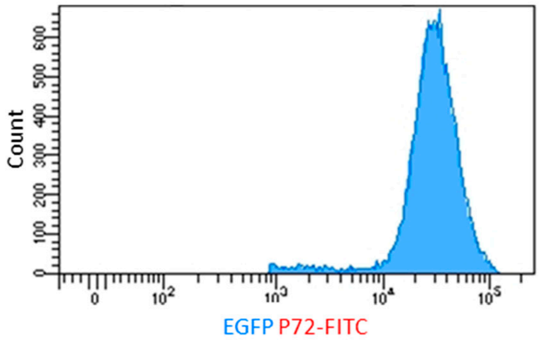

Population	#Events	%Parent	%Total
All Events	20,000	####	100.0
Cells	19,505	97.5	97.5
Singlets	19,418	99.6	97.1
single cells	19,413	100.0	97.1
ASFV+	21	0.1	0.1
EGFP+	18,563	95.6	92.8
P72+	13	0.1	0.1
ASFV+ EGFP+	2	0.0	0.0

WSL GFP
ASFV-infected

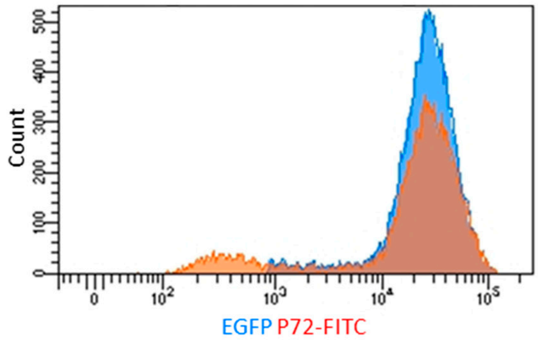

Population	#Events	%Parent	%Total
All Events	20,000	####	100.0
Cells	18,699	93.5	93.5
Singlets	18,418	98.5	92.1
single cells	18,352	99.6	91.8
ASFV+	13,592	74.1	68.0
EGFP+	16,636	90.6	83.2
P72+	12,082	72.6	60.4
ASFV+ EGFP+	8,829	48.1	44.1

WSL GFP-I73R
non-infected

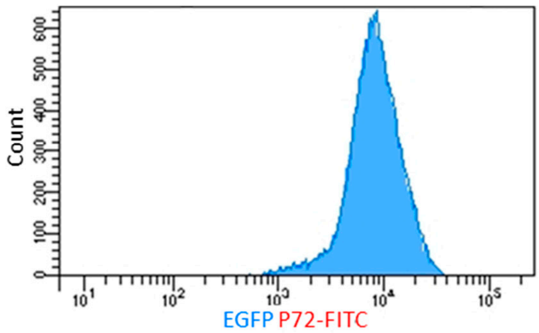

Population	#Events	%Parent	%Total
All Events	20,000	####	100.0
Cells	19,471	97.4	97.4
Singlets	19,264	98.9	96.3
single cells	19,256	100.0	96.3
ASFV+	7	0.0	0.0
EGFP+	19,219	99.8	96.1
P72+	10	0.1	0.0
ASFV+ EGFP+	1	0.0	0.0

WSL GFP-I73R
ASFV-infected

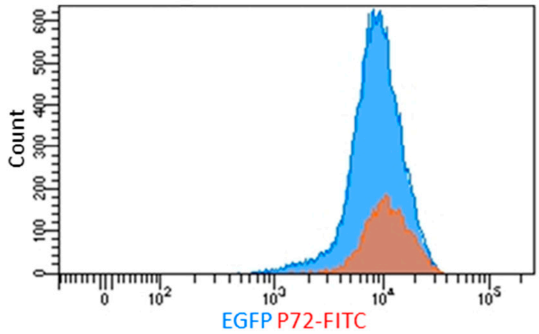

Population	#Events	%Parent	%Total
All Events	20,000	####	100.0
Cells	19,215	96.1	96.1
Singlets	19,053	99.2	95.3
single cells	19,016	99.8	95.1
ASFV+	5,460	28.7	27.3
EGFP+	18,987	99.8	94.9
P72+	5,588	29.4	27.9
ASFV+ EGFP+	4,324	22.7	21.6

Figure S2. Quantification of transfected and infected cell populations. Flow cytometry was utilized to measure the rates of GFP and I73R-GFP transfection and infection in the WSL cell populations. Cells were subdivided based on their expression of the GFP and investigated for ASFV infection with ASFV P72 antibody. The non-transfected WSL cells were used as a control.

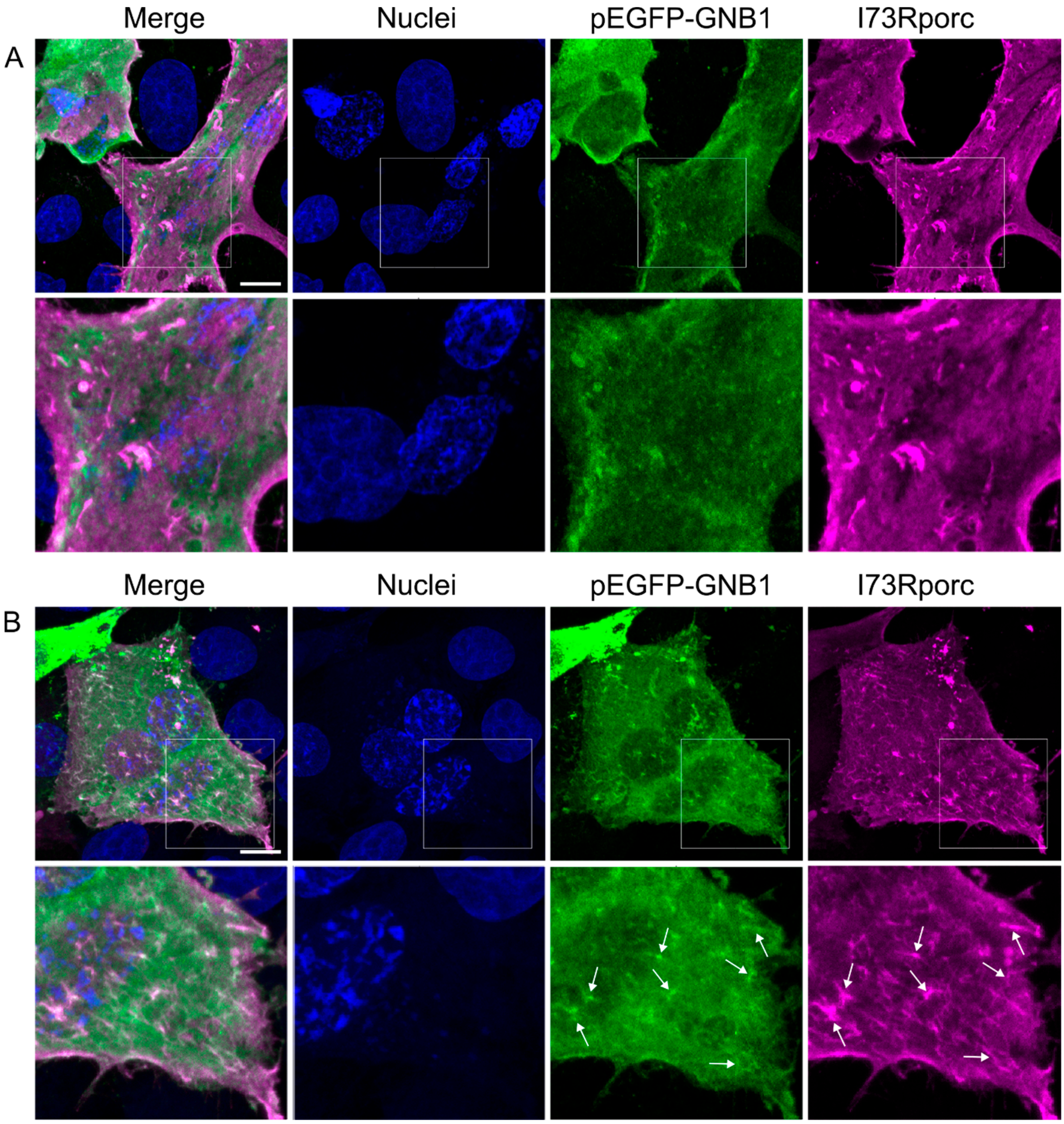

Figure S3. Co-localization of p3xFLAG-I73Rporc and pEGFP-GNB1 in WSL cells studied by confocal microscopy. While no distinct co-localization of p3xFLAG-I73Rporc and pEGFP-GNB1 (A) could be demonstrated in cells with a wide and mostly diffuse staining for both proteins, co-localization was observed in other cells (B) in what appears as cytoskeletal structures (white arrows). Top panels: cell overviews. Bottom panels: zoomed views of the selected areas shown in top panels. Scale bars 10 µm.

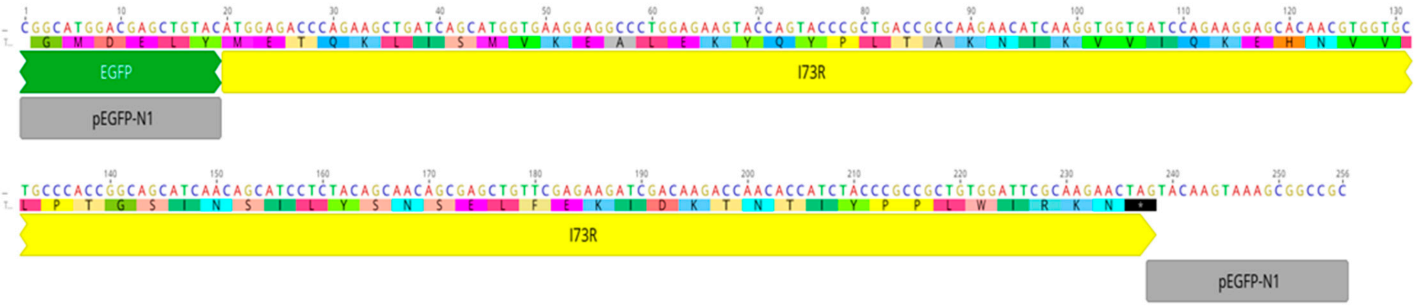

Figure S4. Synthetic ASFV *I73R* gene for expression of fusion protein with EGFP.

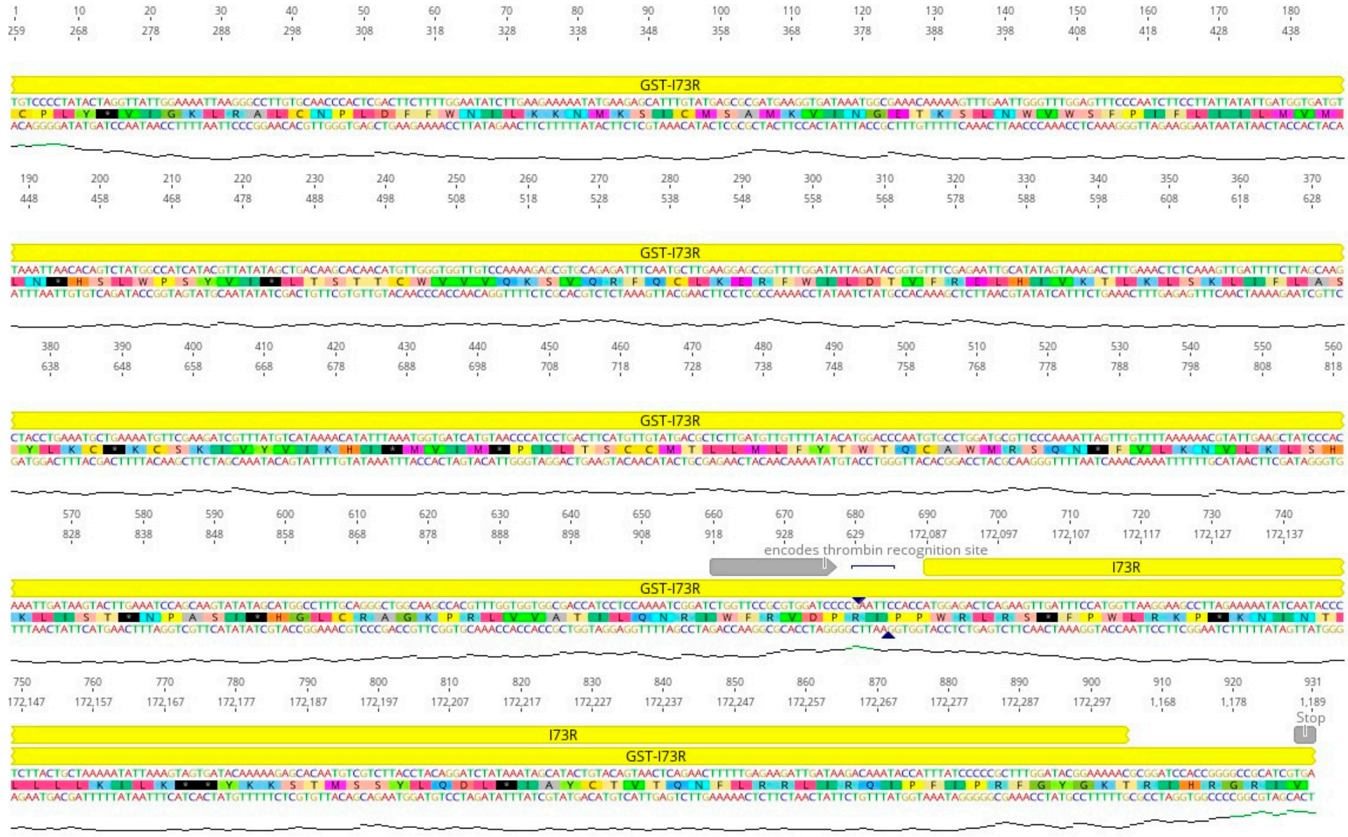

Figure S5. Schematic representation of the GST tag, thrombin cleavage site, and pI73R sequence from ASFV Georgia 2007/1 as cloned into the pGEX-4T3 plasmid.
