# Supplementary material for: Structural and Functional Analysis of ASFV pI73R Reveals GNB1 Binding and Host Gene Modulation
Source: Int J Mol Sci. 2025 Dec 5;26(24):11768. doi: 10.3390/ijms262411768 (PMC12733259; doi:10.3390/ijms262411768)
Supplement: Supplementary file 1 [file ijms-26-11768-s001.zip › Suppl. Methods_I73R_v3-IJMS-submission-final-1.pdf]

Supplementary Materials and Methods

Structural and Functional Analysis of ASFV pI73R Reveals GNB1 Binding and Host Gene Modulation

Katarzyna Magdalena Dolata [1,*], Barbara Bettin [1], Richard Küchler [1], Katrin Pannhorst [1], Dmitry S. Ushakov [1], Walter Fuchs [1], Axel Karger [1,*]

1 Institute of Molecular Virology and Cell Biology, Friedrich-Loeffler-Institut, Federal Research Institute for Animal Health, Südufer 10, 17493 Greifswald-Insel Riems, Germany
* Correspondence: katarzyna.dolata@fli.de (K.M.D.); axel.karger@fli.de (A.K.); Tel.: +49 38351 7 1535 (K.M.D.); +49 38351 7 1247 (A.K.)

S1. Plasmids and DNA transfection

To obtain an expression construct for FLAG-tagged pI73R, plasmid pEGFP-pI73Rporc was linearized with BsrGI, and a 4228 bp fragment of it, lacking the GFP ORF, was amplified by PCR with KOD Xtreme™ Hot Start DNA Polymerase (Merck Millipore) using the primers FLAGI73R-HFF (5′-*AAGGACGATGACGACAAG*CTTATGGAGACC-CAGAAGCTG-3′) and FLAGI73R-HFR (5′-*GGTCCTTGTAGTCCATGGTGG*CGACCG-3′). These primers contained extensions (printed in Italics) overlapping with the synthetic DNA fragment Kozak3xFLAG (5′-*CCACC*___ATGGACTACAAGGACC___**ACGATGGTGATTACAAGGACCATGATATCGACTAC**___AAGGACGATGACGACAAG___-3′) (purchased from Eurofins Genomics) which encoded the 3xFLAG epitope (printed in bold). The overlaps allowed insertion of this fragment into the PCR-amplified vector by ligase-free Hot Fusion cloning [1] In-frame addition of the tag to the 5′-end of I73R was verified by sequencing of the resulting plasmid p3xFLAG-pI73Rporc. An expression plasmid containing the synthetic GNB1 ORF C-terminally fused to GFP in vector pEGFP-C1 was a gift from Dave Piston (Addgene plasmid # 133856) [2].

For colacalization studies WSL cells were cotransfected as described in subsection 4.2 of the Materials and Methods with plasmids pEGFP-GNB1 and p3xFLAGpI73Pporc, or pEGFP-N1 and an expression plasmid for an irrelevant 3xFLAG-tagged protein (p3xFLAGpC) as controls. After two days the cells were fixed for indirect immunofluorescence analyses.

S2. Indirect immunofluorescence (IF) analyses and confocal microscopy

For IF analyses plasmid-transfected WSL cells grown on coverslips were fixed after 2 days with 4% paraformaldehyde in PBS for 30 min, and permeabilized with 0.5% Triton X-100 for 15 min at room temperature (RT). The cells were blocked with 10% FBS in PBS, and coincubated with a GFP-specific rabbit antiserum (kindly provided by G.M. Keil), and a FLAG-specific mouse mAb (F1804, Sigma-Aldrich, Merck Millipore) for 1 h each at RT. Binding was detected by incubation with Alexa Fluor 488 anti-rabbit and Alexa Fluor 594 anti-mouse secondary antibodies (Invitrogen, Thermo Fisher Scientific) for an additional hour, and cell nuclei were stained with 1 µg/ml Hoechst 33342 in PBS for 15 min at RT. After each step the coverslips were repeatedly washed with PBS, and finally mounted onto slides using ProLongTM Glass Antifade Mountant (Invitrogen, Thermo Fisher Scientific). High-resolution imaging was performed with a Leica Stellaris 8 confocal microscope equipped with a HC PL APO CS2 63x/1.40 objective. 1024x1024 pixel image format with 60 nm pixel size was used to match the objective's maximal azimuthal resolution. Confocal z-stacks were recorded at 400 Hz with two line averages.

1. Fu, C.; Donovan W.P.; Shikapwashya-Hasser O.; Ye X.; Cole R.H. Hot Fusion: an efficient method to clone multiple DNA fragments as well as inverted repeats without ligase. *PLoS One*. **2014**, 31;9(12):e115318, doi: 10.1371/journal.pone.0115318.

2. Foust D.J.; Godin A.G.; Ustione A.; Wiseman P.W.; Piston D.W. Two-Color Spatial Cumulant Analysis Detects Heteromeric Interactions between Membrane Proteins. *Biophys J.* **2019**, 117(9):1764-1777, doi: 10.1016/j.bpj.2019.09.028.
